# Post-LASIK dry eye disease: A comprehensive review of management and current treatment options

**DOI:** 10.3389/fmed.2023.1057685

**Published:** 2023-04-11

**Authors:** Atena Tamimi, Farzad Sheikhzadeh, Sajjad Ghane Ezabadi, Muhammad Islampanah, Peyman Parhiz, Amirhossein Fathabadi, Mohadeseh Poudineh, Zahra Khanjani, Hossein Pourmontaseri, Shirin Orandi, Reyhaneh Mehrabani, Mohammad Rahmanian, Niloofar Deravi

**Affiliations:** ^1^Student Research Committee, School of Medicine, Shahid Beheshti University of Medical Sciences, Tehran, Iran; ^2^School of Medicine, Iran University of Medical Sciences, Tehran, Iran; ^3^Students’ Scientific Research Center, School of Medicine, Tehran University of Medical Sciences, Tehran, Iran; ^4^Faculty of Medicine, Mashhad University of Medical Sciences, Mashhad, Iran; ^5^Student Research Committee, Zahedan Medical Sciences Branch, Islamic Azad University, Zahedan, Iran; ^6^Student Research Committee, School of Paramedical Sciences, Mashhad University of Medical Sciences, Mashhad, Iran; ^7^School of Medicine, Zanjan University of Medical Sciences, Zanjan, Iran; ^8^Student Research Committee, Fasa University of Medical Sciences, Fasa, Iran; ^9^Bitab Knowledge Enterprise, Fasa University of Medical Sciences, Fasa, Iran; ^10^Department of Clinical Biochemistry, School of Medicine, Tehran University of Medical Sciences, Tehran, Iran; ^11^Student Research Committee, Semnan University of Medical Sciences, Semnan, Iran

**Keywords:** LASIK, laser-assisted *in situ* keratomileusis, dry eye disease, eye drops, eye disease

## Abstract

Laser-assisted *in situ* keratomileusis (LASIK) is a unique corneal stromal laser ablation method that uses an excimer laser to reach beneath corneal dome-shaped tissues. In contrast, surface ablation methods, such as photorefractive keratectomy, include removing epithelium and cutting off the Bowman’s layer and the stromal tissue of the anterior corneal surface. Dry eye disease (DED) is the most common complication after LASIK. DED is a typical multi-factor disorder of the tear function and ocular surface that occurs when the eyes fail to produce efficient or adequate volumes of tears to moisturize the eyes. DED influences quality of life and visual perception, as symptoms often interfere with daily activities such as reading, writing, or using video display monitors. Generally, DED brings about discomfort, symptoms of visual disturbance, focal or global tear film instability with possible harm to the ocular surface, the increased osmolarity of the tear film, and subacute inflammation of the ocular surface. Almost all patients develop a degree of dryness in the postoperative period. Detection of preoperative DED and committed examination and treatment in the preoperative period, and continuing treatments postoperatively lead to rapid healing, fewer complications, and improved visual outcomes. To improve patient comfort and surgical outcomes, early treatment is required. Therefore, in this study, we aim to comprehensively review studies on the management and current treatment options for post-LASIK DED.

## Introduction

Refractive surgery is a non-invasive method for correcting or improving the refractive state of the eye (1). This surgery has recently become specialized for non-essential and non-sight-threatening conditions (2). Today, ophthalmic surgeons have multiple refractive surgical choices to ameliorate refractive errors (1). In the last two decades, refractive surgery has advanced highly beyond corrective laser therapy. Progress in surgical technologies has also been conducted to introduce intraocular phakic implants, intracorneal implants, and a new minimally invasive refractive surgery approved by the Food and Drug Administration (FDA) (3). The speed and accuracy of laser devices have progressed over the past decade, and the success of laser vision correction is highly related to the precision of these platforms (4).

Refractive surgeries widely divide into refractive corneal lenticular surgery (a kind of stromal ablation that does not need a flap), corneal stromal surgery (making of a corneal flap), and corneal surface ablation surgery (5). Laser-assisted *in situ* keratomileusis (LASIK) is a unique corneal stromal laser ablation method that uses an excimer laser to reach beneath corneal dome-shaped tissues, while surface ablation methods, such as photorefractive keratectomy, include removing epithelium and cutting of the Bowman’s layer and the stromal tissue of the anterior corneal surface (4, 6, 7). On the other hand, other refractive surgeries, such as small incision lenticule extraction (SMILE), suggest a paradigm change in laser vision correction through a less invasive method that creates a lenticule in the intact cornea (< 4 mm) and removes it through an incision (8). These methods’ visual and refractive outcomes have been demonstrated to be similar to LASIK; however, there is some evidence for the advantages of SMILE over LASIK, such as an intact anterior stroma, better biomechanics, and fewer dry eye disease (DED) symptoms and corneal reinnervation (9, 10).

The Bowman’s layer is the cornea’s most vital part, followed by the central tightly interwoven anterior stroma (100–120 μm) (11). Flap thickness in the LASIK group is between 90 and 110 μm, and cap thickness in the SMILE group is between 100 and 120 μm (12). Therefore, the SMILE method removes the deeper stroma, leaving the anterior-most stroma intact and maintaining more structural integrity (13). However, LASIK involves flap creation by cutting the peripheral collagen fibers, thus causing lower stability (12). The lesser inflammatory response in wound healing after SMILE may contribute to better biomechanics (14).

Recently, the morbidity of DED has increased and has demonstrated a younger trend (15). Recent research revealed that DED occurs in up to 87.5% of the global population (mostly in women). On the other hand, the prevalence of DED multiplies every five years after the age of 50 (16). Therefore, the management of LASIK-induced DED (Li-DED) complications attracted much attention in recent studies. Here, we aim to review published studies on Li-DED to understand what approaches can be taken to decrease laser-surgery disorders, especially DED.

## LASIK vs. PRK vs. SMILE

LASIK, the most common method of vision correction treatment, like photorefractive keratectomy (PRK), is used for various types of myopia, hyperopia, and astigmatism cases. SMILE is the newest method of vision correction treatment that is performed for types of myopia and astigmatism.

In the LASIK method, the eye’s cornea is reshaped by creating a thin circular flap on the external tissue of the cornea, and as a result, the incoming light becomes well-focused. PRK is similar to LASIK, with the difference that in this method, instead of using a corneal flap, the upper layer of the cornea, called the epithelium, is removed to expose the inner layer. This method is usually used for patients for whom the LASIK method is unsuitable, such as patients with thin corneas. In SMILE, instead of using an excimer laser, a femtosecond laser creates a small lens-shaped piece of tissue, known as a lenticule, within the cornea. Then, this lens is removed by making an incision in the cornea’s surface. In terms of recovery, the LASIK and SMILE methods are similar, whereas the PRK method requires more recovery time due to the destruction of the corneal epithelium layer.

Few studies have examined PRK and LASIK methods in terms of dry eye parameters; Lee et al. (17) reported a shorter tear break-up time (TBUT) and lower Schirmer test in LASIK than in PRK at 3-month follow-up; however, Bower et al. (18) found more reduction in the Schirmer test in PRK than in LASIK at postoperative 1 and 3 months. On the other hand, several studies have examined SMILE and LASIK methods in terms of dry eye parameters (19–21). The results of a recently published meta-analysis study show that SMILE had better corneal sensitivity, longer TBUT, and lower ocular surface disease index (OSDI) at postoperative 1, 3, and 6 months. However, Schirmer test 3–5 and tear osmolarity 4,5 showed no difference within 6 months of follow-up (20). However, long-term studies have not observed any superiority between these methods (22).

### Post-LASIK complications

Over the past decade, it has been reported that LASIK surgery is the most common vision correction surgery in the world, and this is due to not only high patient satisfaction but also to its rare postoperative complications. However, many patients have complained of dry eye symptoms, namely, irritation, dryness, red eye, and ocular fatigue, the so-called post-LASIK tear dysfunction that disappears over time. Only a few patients complain of these symptoms as long-term problems (23). Although the mechanism involved in post-LASIK eye dryness is not clearly understood, flap creation leads to temporal denervation of the cornea, which causes decreased corneal sensitivity, resulting in this condition. It is reported that corneal sensitivity decreases significantly for 3 months after LASIK surgery, but intracorneal nerves regenerate approximately 3 to 6 months post-operation. Decreased or absent corneal sensitivity is a neurodegenerative disease called neurotrophic keratitis (NK). NK is also known as neurotrophic keratopathy. It is a degenerative corneal disease caused by damage to the trigeminal nerve. Corneal nerves are essential for maintaining normal metabolism, tear production, and ocular surface function. Loss of sensitivity results in loss or reduction of corneal sensation (CS). It can impair corneal wound healing and lead to changes in the epithelium, including persistent epithelial defects, punctate epithelial keratopathy, and corneal ulceration. Due to the low sensitivity of the cornea, patients might have no symptoms such as pain or discomfort in their eyes. However, eye redness and dryness, blurred vision, and decreased clarity of vision might appear (24, 25). NK is classified with an estimated prevalence of fewer than 5/10,000 people (26). Although NK can be stimulated by any condition that affects the nerves serving the cornea, two fundamental causes are the herpes simplex virus and the herpes zoster virus. In addition, surgeries involving the cornea or that are performed around the eye, namely LASIK, cataract surgery, orbital surgery, and corneal transplants, have the potential to damage the cornea, thus leading to NK (25).

The diagnosis of NK requires a careful review of the ocular and systemic clinical history, a thorough eye examination, and an evaluation of corneal sensitivity. Complementary examinations such as *in vivo* confocal microscopy and all diagnostic methods are considered to achieve NK’s correct diagnosis and classification (26). The function of the corneal nerve can be examined and tested quantitatively and qualitatively. Quantitative testing requires the use of a corneal esthetic meter. With the Cochet-Bonnet esthetic meter, mechanical contact is made on the corneal surface with different lengths of nylon thread. The filament is applied to the eye’s surface, and gentle pressure bends the filament. In a qualitative test, dental floss, cotton swabs, or tissue tips stimulate the corneal surfaces bilaterally, and the response is compared in each eye. The response is measured by eliciting the guard blink reflex. The quality of this response can be described as absent from hypostatic to normal. The more significant function of the trigeminal nerve is evident when the patient perceives the cord to be longer. Belmonte’s non-contact gas esthetic meter, which emits carbon dioxide at different temperatures and flow rates, can measure different aspects of corneal sensitivity.

Neurotrophic keratitis (NK) severity correlates with corneal nerve dysfunction during testing. NK is unlikely to occur if the patient has normal corneal sensitivity. However, more work is indicated if the response to eye surface stimuli is reduced (27).

Neurotrophic keratitis (NK) treatment approach should be rapid and based on the severity and stage of the disease. Despite the availability of various medical and surgical treatments, the treatment of NK still needs to be improved, and a lack of positive response is usually observed in clinical practice (25). NK treatment should be based on the severity of the disease. The goal of treating stage 1 disease is to improve the quality and transparency of the epithelium and prevent epithelial breakdown. In the presence of pigment epithelial detachment (PED), treatment is performed to prevent the formation of corneal ulcers and stroma involvement and promote corneal healing. More severe cases with stromal melting and corneal ulceration require immediate attention to prevent perforation and stop stromal lysis (26). In stage 2, the treatment promotes epithelial defect healing and prevents corneal ulcer progression. The patient should be monitored continuously, as progression to asymptomatic perforation and stromal melting may occur. Topical antibiotics are recommended at this stage to prevent infection. Conversely, topical steroids should be used with caution because they may increase the risk of corneal melting and inhibit the healing process. Therapeutic contact lenses improve the cornea, keep the fluid layer in regular contact with the cornea, and protect against eyelid rubbing. However, patients should be careful due to the increased risk of infection (25). An essential feature of stage 3 is the presence of corneal ulceration. Stromal melting may progress to corneal thinning and eventual perforation. This issue may occur without multiple ocular symptoms due to corneal sensitivity disorder. However, patients may complain of blurred vision in edema, corneal ulceration, or scarring (25, 27). Cenegermin (oxervate) is an FDA-approved eye drop for treating NK (28).

Corneal dysesthesia happens when the regeneration of nerve endings results in the exaggeration of stimulus (29–31). However, dry eye signs and symptoms had an incomplete disparity in some observations (32). Although it had been hypothesized that the decreased sensation of the dry eye would ameliorate in some months after the operation due to the regeneration of nerve endings, the slow improvement of thermoreceptors (the nerves involved in the sensation of eye dryness) can explain the reason of this condition (33, 34). In addition, when the osmolarity of the tear increases in patients with preoperative dry eye, the cold thermoreceptors exaggerate the minimal changes after LASIK (33, 35).

After LASIK, some patients experience intense symptoms while they do not indicate any abnormal findings (36). On the other hand, some patients with obvious changes in the epithelium do not complain of any DED symptoms (37). This discrepancy may be due to nervous etiologies rather than tear production (38, 39). Generally, neuralgia is a pain in the nerve distribution with simultaneous nerve damage symptoms. Corneal neuralgia (CN) is a chronic condition in which patients complain of pain and foreign body sensation, often variable and affected by environmental conditions. Persistent DED symptoms following LASIK may be due to CN, which may be a variation of complex regional pain syndrome (CRPS) type 2 (40). Patients with CN indicate poor response to monotherapy with conventional treatments of DED (41). CN is considered to be significantly rare and as a result, its exact incidence is ill-defined (42). This disease is associated with certain comorbidities, such as fibromyalgia, anxiety, depression, headache, HIV, celiac disease, idiopathic small fiber neuropathies, and diabetes. Women have a higher incidence of CN as well as its related comorbidities (43).

The cornea is one of the tissues with the most nervous density. It is among the tissues with the strongest pain sensation. It has virtually 7,000 nerve terminals in each square millimeter. As a result, corneal sensitivity is 300–600 times stronger than skin sensitivity (44). Tactile, temperature, and pain sensations are carried by the nerves of the cornea. Mechanical, chemical, and thermal triggers are identified by these nerves. These triggers are perceived as a form of dysesthesia or pain. Dysesthesia causes unpleasant sensations, including dryness, grittiness, photograph allodynia, irritation, and burning. Pain is a protective mechanism from injury. Nociceptors detect pain stimuli and transmit this signal through action potentials to higher centers for perception. Iatrogenic complications, trauma, and inflammatory states of the ocular surface may impair these nerves, resulting in their hypersensitivity. This sensitization reinforces the signals of pain. Chronic provocation sensitizes the central nervous system and thereby photograph allodynia and higher pain perception occur (43). Peripheral nerve damage might take place during LASIK. CN or corneal neuropathic pain would occur due to a set of adversely regenerated nerve endings after LASIK. The nociceptors become upregulated, bringing about increased responsivity (43, 45).

Patients with CN might report a variety of symptoms, such as aching, boring, burning, foreign body sensation, and photophobia. These manifestations can be debilitating. Blepharospasm may also be present with CN (43). Confirmation of a diagnosis can be achieved through a confocal microscopic view of the cornea, which reveals the abnormal conditions of the nerves. Esthesiometry is another approach to evaluating the response and function of nerve fibers (44). However, these methods are not accessible to all physicians, making CN a diagnosis by exclusion. The identification of CN is very challenging because of a lack of clinical exam signs. Hence, CN can frequently be mistaken for DED (43).

These patients mention increased pain reaction to drops, touch, and air. Cataract or refractive surgery should be taken into account in patient history. Practitioners often disregard the patients’ condition because of scarce clinical findings (38). Normal DED evaluations are initially performed for the patient. The eye surface indicates healthy in CN while patients with DED have impaired tear osmolarity and surface staining (46). Subjective reports of pain without objective DED evidence may be indicative of CN. Of note, DED can be accompanied by CN, which further complicates accurate diagnosis (43).

Severe photophobia and ocular pain can significantly hinder everyday function. Suicidal thoughts can also be present in intense cases (44). A proparacaine challenge test can be used to differentiate between central and peripheral nervous impairment, directing toward the correct treatment option. In this test, 0.5% proparacaine hydrochloride is applied topically. A complete response shows peripheral nerve damage. Additionally, cases with both types show partial response. If there is no alleviation of symptoms, central nerves have been sensitized, making pain management very difficult. There are many proposed therapeutic options for CN (43). Autologous serum can precipitate the regeneration of nerves. Prosthetic replacement of the ocular surface ecosystem (PROSE) may decrease signals of the peripheral nervous system. Topical anti-inflammatory treatments may lower ocular inflammation. Systemic drugs might also hamper or alter pain signals. Neurotrophic factors [especially nerve growth factors (NGFs)] have been reported to reduce pain. Regeneration of impaired nerves of the cornea may alleviate symptoms. In this regard, neurotrophic factors have been evidenced to heal the injured peripheral nerves with improvement in their function. Autologous serum therapy might have the same effect (40).

The prognosis differs for each individual. Chronic CN symptoms usually demand combinational therapy with different methods (38, 44). Early treatment brings about a more encouraging prognosis (43). Regarding CN side effects, anxiety arises in cases with chronic pain and causes even more pain. Furthermore, persistent pain causes physical exhaustion. Depression, fatigue, and joint pain are also found to be associated with CN (43).

All in all, even though post-LASIK dry eye is usually a temporary condition, some severe symptoms are not entirely resolved, which affects patients’ satisfaction and decreases vision quality; therefore, an accurate diagnosis and preventive methods might be effective.

## Dry eye disease (DED)

According to the Tear Film and Ocular Surface Society dry eye workshop II (TFOS DEWS II) definition, dry eye is a disease of the ocular surface caused by different etiological factors. In DED, the tear film homeostasis is compromised with concurrent ocular manifestations. The tear film’s unstable and hyperosmolar state, inflammation, impairment of the ocular surface, and neurosensory dysfunctions contribute to these ocular symptoms (47).

Dry eye disease (DED) is a typical multi-factor disorder of the tear function and ocular surface that occurs when the eyes fail to produce efficient or adequate volumes of tears to moisturize the eyes (47, 48). DED influences quality of life and visual perception, as symptoms often interfere with daily activities, such as reading, writing, or using video display monitors (49). The ocular surface and lacrimal gland are composed of a functional unit where the afferent sensory nerves of the ocular surface and the efferent autonomic nerves to the lacrimal gland help to modulate tear production and secretion. When this interaction is damaged, tear film osmolarity rises and induces corneal epithelium apoptosis and inflammation (50).

Generally, DED brings about discomfort, symptoms of visual disturbance, focal or global tear film instability with possible harm to the ocular surface, increased osmolarity of the tear film, and subacute inflammation of the ocular surface (47, 48). Moreover, DED can result in other symptoms, such as positive vital staining of the ocular surface, receded TBUT and Schirmer test values, decreased corneal sensitivity, ocular dysesthesias, and fluctuating or blurry vision (41, 51).

Although we have several available methods to diagnose and measure DED severity, such as Schirmer’s test, fluorescein break-up time (FBUT), and validated questionnaires, for instance, the OSDI (52), and also reliable therapies to decrease DED complications, such as anti-inflammatory medication, topical ophthalmic drops, autologous serum treatment, and punctual plugs, patients face significant DED symptoms after laser-based operations (53–58). Studies on laser-induced DED provided unalike incidences of this disorder ranging from 20% to more than 50% in patients who underwent LASIK surgery. Patients who underwent SMILE also contracted postoperative DED but the risk of contraction was lower (59).

## Risk factors

Generally, the severity and incidence of Li-DED mostly rely on patients’ characteristics and history. The most significant risk factor for Li-DED is the preexisting experience of DED (60–62). Studies on laser-induced complications showed that ocular surface recovery after LASIK is affected by preoperative tear volume (63). Edward et al. (64) reported that patients with less than 10 mm in the Schirmer test before the operation were more susceptible to DED induced by LASIK. Moreover, refractive surgery, women (especially in premenopausal ages), postmenopausal estrogen therapy, and older age increase the risk of incidence and severity of Li-DED (65, 66). Those of Asian origin are also more at risk of developing DED. Increased rates of attempted refractive corrections, dynamics of blinking, tear film parameters, and anatomy of orbit and eyelid in Asian eyes promote DED incidence in Asian populations (67). A genetic effect with polymorphism in the thrombospondin-1 gene can also cause chronic inflammation of the ocular surface (68).

Several studies in the last decades showed the importance of comorbidities in DED complications. Collagen vascular diseases, such as seronegative spondyloarthropathies, systemic lupus erythematosus, Sjogren syndrome, and rheumatoid arthritis, are the other risk factors that usually result in Li-DED (69). Other factors that may facilitate the development of Li-DED are diabetes mellitus, previous blepharoplasty, and lagophthalmos (62, 70).

The parameters used in the LASIK surgery (including the ablation zone and suction time) can induce DED (71, 72). The ablation depth and thickness of the flap are the other surgical factors that cause DED through corneal nerve transection (73, 74). Besides, patients that require more refractive correction are more prone to developing DED (75–77). Patients who undergo LASIK for high myopia, from –9.10 to –14.00 D, can experience DED a few years after the operation (76). On the other hand, a thinner flap may preserve blink rate and CS and diminish corneal nerve invasion, reducing Li-DED duration and severity (78).

At an early post-LASIK stage, ablation depth is associated with densities of the corneal nerve or meniscus of the tear; however, at later stages, goblet cell destiny can affect Li-DED (79). Moreover, wearing contacts for long periods results in delayed recovery of tear secretion, pro-inflammatory cytokines elevation, and reduced corneal sensitivity (80–83). Ocular allergy may also contribute to Li-DED (84).

Surgical changes to the ocular surface often induce Li-DED. Several studies demonstrated that inflammation induced by altered levels of cytokines, damage to the corneal afferent nerve with resultant hypoesthesia, reduced production of tears, distribution of abnormal tears from changes in corneal contour, reduced rates of blinks, and loss of conjunctival goblet cells by the suction ring of LASIK remarkably increase DED severity (28, 85, 86). Sensory denervation instigated by excimer photoablation and transection of nerve throughout the flap creation can lead to Li-DED (87). In addition, some studies indicated a correlation between corneal nerve impairment and Li-DED (87, 88).

Hinge position is another risk factor for Li-DED because most corneal nerves go into the stroma in the horizontal meridians (60). Feng et al. (89) showed that the horizontal hinge flap improved TBUT, corneal sensitivity, and corneal fluorescein staining 3 months after LASIK compared to horizontally hinged flaps. However, the same after 6 months.

## Flap/hinge characteristics

The corneal sensory nerves have a crucial role in tear production, initiating the blink reflex and keeping the corneal epithelium alive (74). Since corneal nerves are meant to be injured during modern refractive surgeries, the corneal innervation subject has been brought to attention (90). Although LASIK disrupts corneal innervation, it has become one of the most reliable refractive surgeries in the last decades (90, 91). It seems that decreased CS due to this disruption may be determinative in the Li-DED condition (90, 91).

There have been two models of corneal innervation proposed. Some studies indicate a dominancy of corneal nerves entering the eye from the medial and lateral rather than superior or inferior sides (92). Using *in vivo* confocal microscopy, other models suggest an inferior-superior orientation of sub-basal nerve fibers with other leashes in different directions (93). As there is variability in the patterns through which corneal nerves run into the cornea, changing flap characteristics such as flap thickness, hinge position, hinge width, and hinge angle during LASIK surgery may have different outcomes in postoperative CS and DED (94).

To date, many studies have been trying to figure out the association between flap/hinge characteristics and Li-DED (74, 90, 91, 94–100), and while studies suggest a relationship between flap/hinge characteristics and Li-DED, others mention no such relationship.

The pros for this relationship would be divided into hinge position, hinge width, and flap thickness. DED is more prominent in eyes with a superior hinge flap than those with a nasal hinge flap (95, 96). Besides, other studies showed that eyes with nasal hinge flaps usually demonstrated better CS recovery (96, 97). In contrast, De Paiva et al. (99) reported that nasal hinge flaps show more reduction in CS than the superior hinge flaps one week after surgery, although CS returned to comparable preoperative levels at 6 months.

Studies on hinge width showed that in wider hinge flap eyes, patients had a better CS, especially 1 and 3 months after surgery, and less DED (74). Moreover, DED is correlated with the degree of preoperative myopia, the laser ablation depth, and flap thickness (99). A thicker flap might increase the volume of nerve regeneration and thereby slow neurosensory corneal recovery (94). Several studies have reported thinner flaps to cause a significantly lower rate of CS loss in microkeratome and femtosecond laser LASIK (97). Another study suggested faster DED healing in thinner flaps with higher Schirmer test scores and longer TBUT when investigating 130 μm and 100 μm flaps. Nonetheless, the other DED parameters were the same, and both groups experienced mild symptoms, which might suggest the existence of a threshold (greater than 130 μm) for flap thickness regarding significant differences in DED symptoms (94).

Tai and Sun (101) conducted a comparative study, investigating the effects of flap diameter in 113 eyes. Interestingly, the results indicated that flap size alone could not be regarded as a single determinant of DED. There was no critical difference regarding all DED parameters between different flap size groups. However, the strong association of the ratio of the diameter of the flap/cornea with DED was noted. The group with a larger flap/cornea diameter ratio experienced decreased Schirmer test and longer TBUT than the other group. This suggests the importance of individualized flap diameter measurement for surgeons (101).

In addition, the angle of flap side cuts is another factor that may affect post-LASIK DED. Evidence suggests that flaps with inverted side-cuts improved the process of wound healing and CS compared to conventional side-cut flaps (102, 103). One prospective study with 120 eyes reported better corneal neurosensory recovery in the eyes with inverted side cuts (130°) than with standard traditional side cuts (70°) at 1, 3, 6, and 12 months after surgery. Nevertheless, the subjective DED symptoms were not notably different in the eyes with different side-cut angles at any follow-up time points (104).

Several studies reported that the hinge position did not affect CS or DED symptoms (91, 98, 100). Vroman et al. (90) suggested no difference between these two groups in the occurrence of DED (although the nasal hinge group significantly had a better nasal CS at 1 month). In another study, Mian et al. (94) showed that hinge position, hinge angle, and flap thickness did not affect CS or DED.

Therefore, flap/hinge characteristics may affect CS and Li-DED, but in most cases, the long-term results are not significantly different between the two groups (74, 90, 91, 94, 97–100). Further studies on corneal innervation maps and regrowth patterns would play an essential role in determining the connection between these two procedures and developing reliable methods to minimize Li-DED. Besides, more expanded studies on some other known risk factors, such as preoperative neurotrophic corneas or DED, and also pre/post-surgery actions taken into consideration are required to achieve the best results in the future (Table 1).

**TABLE 1:** Details of the reviewed studies.

References	Intervention	Outcomes	Setting	Patients (eyes)	Keratome/laser type(s) (flap creation)	Laser type(s) (stromal ablation)	Flap/hinge technical characteristic	Tests used	Follow-up intervals
Lee et al. (95)	Hinge position	RR and DES	South Korea, same center	30 (50)	• Hansatome (Bausch & Lomb Surgical) or the M2 (Moria): superior hinge (25) • SCMD (New United Development Corp.) or the Summit Krumeich-Barraquer (SKBM, Alcon Surgical): nasal hinge (25)	Star S2 excimer laser (VISX)	• Flap thickness: • 160 μm • Flap diameter: • 8.5 mm	• BUT • ST (without anesthesia)	2 months
Donenfeld et al. (96)	Hinge position	CS and DES	USA, same center, same surgeon	52 (104)	• Hansatome microkeratome (Bausch & Lomb, Rochester, NY, USA): superior hinge • Amadeus microkeratome (Allergan Pharmaceuticals, Irvine, CA, USA): nasal hinge	VISX Star II excimer laser (VISX, Inc., Santa Clara, CA, USA)	• Flap thickness: • 160 μm • Flap diameter: • 9.5 mm	• Masked Cochet–Bonnet esthesiometry • LGS (corneal and conjunctival) • ST (with anesthesia) • TBUT • Subjective evaluation of DE sensation	1 week 1 month 3 months 6 months
Donenfeld et al. (74)	Hinge width	CS and DES	USA, same center, same surgeon	54 (108)	• Amadeus microkeratome: nasal hinge (horizontal flap)	VISX Star 3 excimer laser	• Flap thickness: • 160 μm • Flap diameter: • 9.5 mm • Hinge widths: • 0.6 mm vs. 1.2 mm	• Cochet-Bonnet esthesiometry • LGS (corneal and conjunctival) • ST (with anesthesia) • TBUT	1 week 1 month 3 months 6 months
Nassaralla et al.(97)	Hinge position and flap thickness	CS	Brazil, Same center, Same surgeon, 1 (right) eye of each patient	40 (40)	• Hansatome microkeratome: superior hinge • ACS microkeratome: nasal hinge	Technolas 217-C Excimer laser (Bausch & Lomb, Rochester, NY, USA)	• Flap thickness: 160 and 180 μm (Sup. H) 130 and 160 μm (Nasal H) (four groups of eyes) • Flap diameter: 8.5 mm	• Cochet-Bonnet esthesiometry	Q1 month until full recovery
Ghoreishi et al. (99)	Hinge position	DES	Iran, same center, same surgeon	106 (212)	• Hansatome zero compression • Microkeratome (Bausch & Lomb, Rochester, NY, USA): both hinges	Technolas 217 z excimer laser (Bausch & Lomb)	• Flap thickness: • 160 or 180 μm • Flap diameter: • 8.5 or 9.5 mm ü All are determined by the surgeon (based on each individual) but the same in both eyes	• Visual acuity • Fluorescein tear-film breakup time • ST (with anesthesia) • OSDI questionnaire (subjective)	1 week (except OSDI) 1 month 3 months 6 months
Vrom an et al. (90)	Hinge position	CS and DES	USA, same center	47 (94)	• Hansatome microkeratome (Bausch & Lomb): superior hinge • Amadeus microkeratome: nasal hinge	VISX S3 laser	• Flap thickness: 180 μm in SH Flap 160 μm in NH Flap • Flap diameter: • 8.5 mm • Hinge width: • 5.0 mm	• Visual acuity • Contrast sensitivity • CS (Cochet-Bonnet corneal esthesiometer) • Basic secretion test • TBUT • OSS (conjunctival and corneal) • OSDI questionnaire (subjective)	1 week 1 month 3 months 6 months
De Paiva et al. (99)	Hinge position	CS, DES, visual acuity, and ocular surface parameters	USA, same center, three surgeons for surgeries but one surgeon for follow-up, bilateral LASIK and same flap in both eyes of each patient	35 (70)	• Hansatome microkeratome (Bausch and Lomb, Rochester, NY, USA): superior hinge • Amadeus microkeratome (Advanced Medical Optics, Irvine, CA, USA): nasal hinge	VISX Star IV excimer laser (VISX Inc, Santa Clara, CA, USA)	• Flap thickness: • 160 μm • Flap diameter: • 9.5 mm	• Corneal surface evaluation • Optical aberrometer • Snellen visual Acuity measurement • TBUT • CFS • Belmonte modified noncontact gas esthesiometry (for corneal sensitivity) • ST	1 week 1 month 3 months 6 months
Mianet al. (91)	Hinge position	CS and DES	USA, same center, university-based academic practice	33 (66)	• 30 KHz IntraLase Femtosecond Laser (IntraLase Corp.): both hinges	Technolas 217 excimer laser (Bausch & Lomb)	• Flap thickness: • 130 μm • Flap diameter: • 9.0 mm • Hinge angle: • 45 degrees	• Central cochet-bonnet esthesiometry • OSDI questionnaire (subjective) • ST (with anesthesia) • TBUT • CFS • Conjunctival LGS (with a modified Oxford scale)	1 week 1 month 3 months 6 months 12 months
Mian et al. (94)	Hinge position, hinge angle and flap thickness	CS and DES	USA, same center, university-based academic practice	95 (190)	• 30 or 60 KHz IntraLase FS laser (IntraLase Corp.): both hinges	Technolas 217 excimer laser (Bausch & Lomb)	• Flap thickness: • 100 or 130 μm • Flap diameter: • 9.0 mm • Hinge angle: • 45 or 90 degrees	• Central cochet-bonnet esthesiometry • OSDI questionnaire (subjective) • ST (with anesthesia) • TBUT • CFS • Conjunctival LGS (with a modified Oxford scale)	1 week 1 month 3 months 6 months 12 months
Huang et al. (100)	Hinge position	CS and DES	N/A	43 (86)	• Femtosecond laser	N/A	• superior- versus nasal-hinged flaps	• Corneal esthesiometry • BST • TBUT • OSS • OSDI questionnaire (subjective)	1 week 1 month 3 months 6 months

RR, refractive results; DES, dry eye syndrome; SCMD, standard cubic meters per day; TBUT, tear break-up time; ST, Schirmer test; CS, corneal sensation; LGS, lissamine green staining; DE, dry eye; ACS, automated corneal shaper; OSID, ocular surface disease index; OSS, ocular surface staining; CFS, corneal fluorescein staining; BST, Schirmer basic tear secretion test.

## Presurgical evaluation

Before LASIK, patients with refractive disorders pass a complete evaluation of DED and the ocular surface. The most crucial preoperative evaluation is the Schirmer testing, tear quality, presence of lid disease, TBUT, tear meniscus, and any ocular surface staining (105, 106). Moreover, feelings of dryness, grittiness, fluctuations of vision (particularly in conjunction with blinking), and tired eyes are the other vital symptoms in the clinical process of DED (107).

The complaints of blurry vision by patients with cataracts should be differentiated from DED-associated fluctuating blurriness. This information can be collected by utilizing questionnaires, including the impact of dry eye on everyday life (IDEEL) and the OSDI (108, 109). Other examinations for DED are lactoferrin measurement, tear osmolarity, goblet cell count, tear lysozyme measurement, and tear mucin measurement (110). Corneal fluorescein staining assesses the corneal epithelial defects. Anterior segment spectral-domain optical coherence tomography is used to assess the tear meniscus. Corneal confocal microscopy, a less invasive method (111), measures the density of the sub-nasal nerve and evaluates corneal wound healing. Patients diagnosed with persistent corneal staining or reduced vision because of external ocular surface disease and severe DED should not undergo LASIK surgery (112).

Before surgery, external ocular surface abnormalities should be treated (107). Patients with a high risk of DED should begin a regimen and control their therapeutic efficacy. In the pre-surgery term, all patients should be aware of enough lubrication of the external ocular surface. Punctal plugs decrease the drainage rate of tears preoperatively. Moreover, corticosteroids or cyclosporine eyedrops treat dry eye inflammatory components before surgery (113). Ultimately, Lid margin disease should be treated before operation (107).

In terms of medical history and demographic data, it has been reported that women, older age, long-term contact lenses use, having a lower preoperative best-corrected visual acuity (BCVA), and lower preoperative refractive error are associated with higher risks of developing DED within 6 months after surgery.

As a person ages, the sensitivity of the cornea decreases, increasing the risk of developing DED. However, there have been controversies regarding the effect of age on developing DED after surgery. Additionally, some discrepancies have been reported on the association between refractive correction, ablation depth, and postoperative DED, which can be due to differences in the criteria defining DED (50). As for contact lenses, wearing them for a long time increases proinflammatory cytokines and tear film instability and decreases corneal sensitivity.

Some diseases have been suggested to be associated with developing DED postoperatively. Collagen vascular diseases, including systemic lupus erythematosus, rheumatoid arthritis, Sjogren’s syndrome, and seronegative spondyloarthropathies, have established ocular complications such as DED. However, some studies have shown good postoperative results and minimum ophthalmic complications in well-controlled patients (84).

Considering neural disorders, reduced corneal sensitivity is associated with post-LASIK DED. Also, a loss of neural stimulation decreases aqueous tear production, thus, increasing ocular surface inflammation and tear osmolarity. Incomplete blinking and reduced blink frequency increase tear evaporation, exacerbating aqueous production deficiency (114).

In essence, the clinician should be fully prepared to perform surgery, assessing the patient with three tools: history taking, clinical examination, and a short period of pre-surgical treatment. Among these, the most practical, available, and effective examinations and tools are mentioned in Table 2.

**TABLE 2:** Preoperative DED assessment.

History/symptoms	IDEEL OSDI	
Physical examination/signs	Slit lamp based	Eyelid examinations Tear examinations (volume and debris) Injection and staining procedures
	TBUT OPI Schirmer test	
Pre-surgical treatment	Treatment may include artificial tears, ocular steroids, punctal plugs, lid hygiene regimens, etc.	

IDEEL, impact of dry eye on everyday life; OSDI, ocular surface disease index; TBUT, tear break-up time; OPI, ocular protection index.

## Differences regarding surgery methods

Popular refractive surgery includes microkeratome (MK), femtosecond laser (FS), and SMILE. Li-DED incidence is more significant in MK than in FS (71). In addition, FS causes a more prominent reduction in the population of goblet cells than MK due to the difference in the mean period of suction ring activity on the conjunctiva (71, 90). FS has thinner flaps, leading to fewer Li-DED and less afferent sensory damage in the anterior stroma (71, 90). In a novel study, Xia et al. (115) showed that MK and FS for LASIK flap cutting correct myopia, with no remarkable difference throughout 6 months of follow-up. They indicated that MK and FS are safe for LASIK flap cutting, and refractive outcomes stayed stable after a month post-operation. They also showed that FS has more advantages in longer TBUT, predictability of flap thickness, and better cerebero spinal fluid (pressure) (CSF) (115).

In another study, Salomão et al. (71) reported that FS flaps have fewer Li-DED than MK ones, and also required less treatment compared with MK. The reason behind this condition is that FS generates thinner flaps. Thus, afferent sensory nerves have less damage in the anterior stroma. Since FS causes more mean spherical equivalent correction than MK, more Li-DED and more ablation of the stromal nerve are expected in FS. However, MK has a Li-DED incidence. Sun et al. (116) stated that MK and FS increase OSDI scores and corneal staining but decrease TBUT and CS. However, post-operation TBUT is remarkably higher in FS.

On the other hand, Golas and Manche (66) indicated no remarkable difference in self-reported DED symptoms between FS and MK. Several studies showed that SMILE is correlated with a higher score in the DED questionnaire, reduced corneal epitheliopathy risk, elevated Schirmer test value, faster nerve growth factor, lower interleukin-6, less CS and sub-basal nerve density reduction, and longer TBUT in comparison to FS and MK (117–123). SMILE corrects myopia and myopic astigmatism. Cai et al. (124) determined that, mainly within 3 months, recovery of dry eye is better in SMILE than FS and that, in the first 3 months post-surgery, the cornea’s sensitivity recovers better in SMILE than in FS.

## Treatment approaches

### Cyclosporine A

Cyclosporine A is a calcineurin-inhibiting anti-inflammatory peptide that exerts its immunosuppressing effects by preventing T-lymphocyte activation, leading to the restriction of inflammatory cytokine production. This agent is produced as a metabolite of the fungi *Beauveria nevus* and *Tolypocladium inflatum* (114–116). Topical use of cyclosporine A enhances goblet cell density and tear generation (125). Cyclosporine A also blocks the permeability transition pores of the mitochondria and subsequently hinders apoptosis (126). In addition, this agent is regarded to increase corneal sensitivity by seemingly inducing the reproduction of corneal axons (125).

Cyclosporine A is implemented as an FDA-approved agent for many inflammation-associated diseases, including persistent nummular keratitis, ocular inflammation, and ocular-inflammation-induced DED (54, 127). Several clinical studies have confirmed the efficacy of topical cyclosporine A (0.05% emulsion) in treating Li-DED (54, 128–130). Moreover, cyclosporine A is more beneficial in treating DED patients than artificial tears and diquafosol (128, 131). However, temporary pain, irritation, lacrimation, and sensation of a foreign body are seen to be the local complications caused by the topical use of this drug on the eye (131). Supplementary Table 1 summarizes the potential treatments for DED.

### Diquafosol

Another topical drop employed in Li-DED has been diquafosol tetrasodium solution (132, 133). Diquafosol activates P2Y2 receptors and elevates intracellular Ca2+ to promote fluid conveyance by epithelium cells, conjunctiva mucin production by goblet cells, and the provocation of lipid generation (134, 135). Diquafosol (3% solution) has indicated promising outcomes in the DED treatment of different entities, including cataract-surgery-associated, LASIK-associated, contact-lens-associated, aqueous deficiency, meibomian gland dysfunction (MGD), TBUT, and computer-display-use-associated DED (136).

Both monotherapies with diquafosol and their combination with sodium hyaluronate have demonstrated encouraging therapeutic benefits in treating Li-DED (132, 133). Nevertheless, a combination regimen of diquafosol with sodium hyaluronate appears more efficacious in improving DED symptoms and tear production (132). Major adverse complications of this drug in DED include irritation, discharge, conjunctiva injection, pain, pruritus, foreign body sensation, and eye discomfort. Most of these adverse reactions emerged in modest intensity, were reversible, and were eventually eliminated. Hence, diquafosol is considered to have no significant drawbacks (137, 138) (Supplementary Table 1).

### Artificial tear therapy

Artificial teardrops are a traditional treatment for DED that help reduce symptoms (139). Ingredients of artificial tears are composed of two main parts: emollients and demulcents. Emollients are natural fats or oils, such as flaxseed oil or castor oil, which are used to increase the thickness of the fat layer and thus maintain the stability of the tear film and reduce evaporation. Demulcents are water-soluble polymers that help maintain and lubricate the eye’s mucous membrane. The most common of these is carboxymethylcellulose (CMC), which increases viscosity and more extended corneal coverage. In the composition of many artificial tears, in addition to the above (emollient-demulcents), preservatives, such as benzalkonium chloride, polixetonium, polyquaternium (Polyquad), and OcuPure, reduce the growth of bacteria.

Several studies have shown that artificial tears significantly improve DED symptoms compared with placebo (140–143). However, the variety of artificial tears and the lack of randomized clinical trials to compare different types of artificial tears make it difficult to determine which combination is superior to the other (144). Investigations have also caused side effects for this treatment, the most common of which are blurred vision, foreign body sensation, eye discomfort, and irritation (140, 142, 145–147) (Supplementary Table 1).

### Autologous serum

Tears are antimicrobial, mechanical, nourishing, and optical components. They include many active components, such as fibronectin, growth factors, and various vitamins, to provide growth, differentiation, and migration of both conjunctival and corneal epithelium. The absence of these epitheliotropic components in some diseases, such as Li-DED, leads to more severe side effects on the ocular surface (148). Autologous serums are blood derivatives in the shape of eye drops that are naturally non-allergic and biomedically similar to natural tears (148, 149).

IV cell culture and many clinical cohort studies showed that the function and morphology of epithelial cells in the cornea are noticeably preserved (148). In addition, many studies proved the powerful therapeutic effect of the serum on accelerating the curative response and reducing symptoms in moderate to severe DED. This enhancing impact is induced by bioactive proteins and growth factors produced, synthesized, and available in the blood, mainly by activated alpha-granules of platelets (55, 149, 150). New studies aim to evaluate the effectiveness of autologous E-PRP (platelet-rich plasma eye drop) monotherapy of post-LASIK chronic ocular surface syndrome (OSS) in large numbers of cases. In the short term, autologous serum combined with artificial eye drops showed more promising outcomes (151). However, autologous serum therapy had no significantly different outcomes compared with artificial eye drops, but autologous serum results showed fewer adverse effects (152). In a prospective controlled randomized study, E-PRP as a postoperative LASIK treatment was beneficial for improving epithelial healing. However, it appeared to have no “extra” positive effect on corneal nerve regeneration or sensitivity. In addition, recent trials introduced autologous platelet-rich plasma as a novel therapy for patients with severe DED, which performed better than autologous serums (153).

Although a positive serology induced some potential complications, such as scleral vasculitis in some patients with rheumatoid arthritis and some immune-induced inflammatory response correlated with the circulating of antibodies in the serum and antibodies of the cornea, this method has no other serious complications (148, 154–156). However, the abovementioned complications could have happened in the natural course of rheumatoid arthritis (148) (Supplementary Table 1).

### Growth factors

The NGF is a binding polypeptide that adjusts the growth and survival of mature neurons in the nervous system (157). It has become known as an active mediator in many vital functions in mammals’ central nervous system (158). It also performs as a differentiating and survival factor for sympathetic neurons and neural crest sensory (1). NGF induces neurite outgrowth by neuronal cells and reinstates the function of damaged neurons (157, 159).

In a previous study, Bonini et al. (160) indicated that topical NGF eyedrops develop corneal sensitivity and the healing process of corneal epithelial in patients with severe and moderate NK. The assessment of the ocular surface following LASIK condition in patients after prescribing plasma rich in growth factor (PGRF) or artificial tears notably improved all clinical variables (except for best-corrected visual acuity or BCVA). These findings propose the possible effectiveness of PGRF eyedrops in preventing Li-DED compared with conventional eyedrops. Additionally, these results have shown that improving dry eye syndrome PGRF is a lot more effective than conventional treatment. Hence PGRF is suggested for patients suffering from postoperative DED (56) (Supplementary Table 1).

### Punctal plugs

Punctal plugs provide a safe procedure to ameliorate moderate and severe DED (161, 162). They are divided into several types based on their duration of placement (short-term, long-term, or permanent placement) or material (silicone, collagen, hydrogel, polydioxanone, and acrylic) (57, 161). Short-term plugs are usually made of collagen (collected from animals) and can be used for 4–14 days. On the other hand, long-term plugs are made of silicone, hydrogel, polydioxanone, and acrylic and last up to 6 months. Therefore, long-term plugs provide a better choice for postoperative terms (161). Since laser-related DED is temporary after laser procedures, such as LASIK, punctual plugs are urged (162).

The surgeons diminish tear drainage by inserting punctal plugs into the inferior eye punctual opening (161–163). The insertion can be performed at the beginning of the operation and after it (57, 163). This occlusion boosts and maintains natural tears and eye lubricants (57). Although numerous articles have supported the efficacy of punctual plugs in treating DED, some complications, such as allergic reaction, canaliculitis, migration, chronic irritation, conjunctivitis extrusion, and epiphora, can develop from this procedure (57, 161–167) (Supplementary Table 1).

### Omega-3 fatty acids

Omega-3 fatty acids (v3FA), including eicosapentaenoic acid (EPA), docosahexaenoic acid (DHA), and alpha-linolenic acid, are essential fatty acids for body metabolisms. Since these essential fatty acids cannot be synthesized in our bodies, we have to obtain them through our diet. Upregulation of NGF secretion is induced by injury to the cornea (168). In animal models, regeneration of post-injury nerve to the cornea in the eye is improved by DHA plus pigment epithelial-derived factor (PEDF) combinations and DHA plus NGF combinations (169, 170). Therefore, supplementation with v3FA would play an essential role in LASIK.

Several studies demonstrated that high v3FA consumption also causes a significant proportion of these accumulations in cell membrane phospholipids. The v3FA substrates-generated eicosanoids include thromboxane (TX) A3 and leukotriene (LT) B5. They have an anti-inflammatory role against arachidonic acid-generated eicosanoids, such as LTB4, PG E2, and TX A2 (arachidonic acid-generated eicosanoids have a pro-inflammatory role) (171–173). Moreover, v3FA administration improves goblet cell density in patients with DED (174, 175). Therefore, v3FA positively affects the goblet cells, regenerates nerves, and has an anti-inflammatory role in Li-DED pathophysiology (Supplementary Table 1).

### Eyelid-warming devices

The efficacy of many types of warming devices on tear function and the ocular surface have been considered and these devices have been divided into “non-wet warming” and “wet warming” according to the remaining water on the lid surface after or during warming (176). Eye warmer, a disposable eyelid-warming device based on iron oxidation, significantly increases eyelid temperature and melts the meibomian gland lipid. Thus, MGD can be treated (177). Blephasteam, an eyelid-warming device, provides humidity and warmth for eyelids to liquefy meibum and decreases conjunctival hyperemia and ocular symptoms, including sensitivity to light and burning sensation (178). Di Pascuale et al. (179) used eye-feel, an eye-warming device that focuses on the role of lipids in tear insufficiency-1480408721-1480408721, for the treatment of persistent Li-DED (Supplementary Table 1).

### Thermal pulsation

After prevalent therapies such as omega-3 supplementation, eyelid hygiene, and hot compresses, thermal pulsation is now the primary treatment for evaporative DED and MGD (180). Vectored thermal pulsation (VTP) can transfer heat by overcoming obstacles and discharging the gland’s contents. It is done by a device, called Lipiflow, that heats (42.5°C) the interior of the upper and lower eyelids to insulate both eyes and concurrently apply pressure by distending and compacting an inflatable air bladder to the outer of the eyelids. This device should be used by physicians only in adult patients (180, 181) (Supplementary Table 1).

Some studies confirm the efficiency and safety of thermal pulsation for treating dry eye disease by improving the TBUT after laser vision correction and refractive surgery (181–183). Adverse events of the device were temporary, predictable, and not serious (180).

### Intense pulsated light

The Intense Regulated Pulsed Light is a polychromatic pulsed light generator that can produce homogeneous and calibrated light impulses (184). The sculpted pulses are released in the form of pulse trains whose distance, energy, and spectrum are precisely determined to stimulate the meibomian glands and make them return to normal function (184). Wavelengths of light ranging from 400 to 1,200 nm generated by xenon flashlamps of intense pulsated light (IPL) and are limited to visible light by a filter (185). Fuentes Páez et al. (186) suggested that IPL can reduce symptoms and signs of chronic evaporative DED in patients who sustain LASIK surgery; however, more studies are required with more patients and longer follow-up times. Swelling, light sensitivity, floaters, blistering, and facial redness have been reported, but they are not earnest adverse events by the definition of the FDA (185) (Supplementary Table 1).

### Acupuncture

Acupuncture is an old Chinese treatment that has been effective for some diseases, such as ophthalmologic diseases (187, 188). Previous studies showed that acupuncture diminishes Li-DED symptoms (189) and that it is almost better than artificial tears for DED treatment (188). Acupuncture increases the secretion of the lacrimal gland and ameliorates DED through stimulation of the immune system and autonomic nerves (187). There are limited studies on the effects of acupuncture on DED after refractive surgery, and it is necessary to research more about the physiology of this condition (187). Few studies reported on the side effects of this treatment method. For example, Kim et al. (190) reported three cases of hematoma with different intensities. Jang et al. (189) described local, general, and psychological adverse events that may be related to acupuncture (Supplementary Table 1).

## Other studied treatments

### Prosthetic replacement of the ocular surface ecosystem (PROSE)

Prosthetic replacement of the ocular surface ecosystem is a type of FDA-validated contact lens fabricated from a fluorosilicone-acrylate polymer that is permeable to gas. These scleral lenses are custom-made by computer software for each patient’s eyes. This device has indicated efficacy in ocular surface diseases and intense ectasia (191, 192). Scleral lenses save the patient’s eyesight when every other therapy method has failed (193). PROSE devices have been proven advantageous for patients with Li-DED (194) (Supplementary Table 1).

### Pregabalin and gabapentin

**FIGURE 1:**
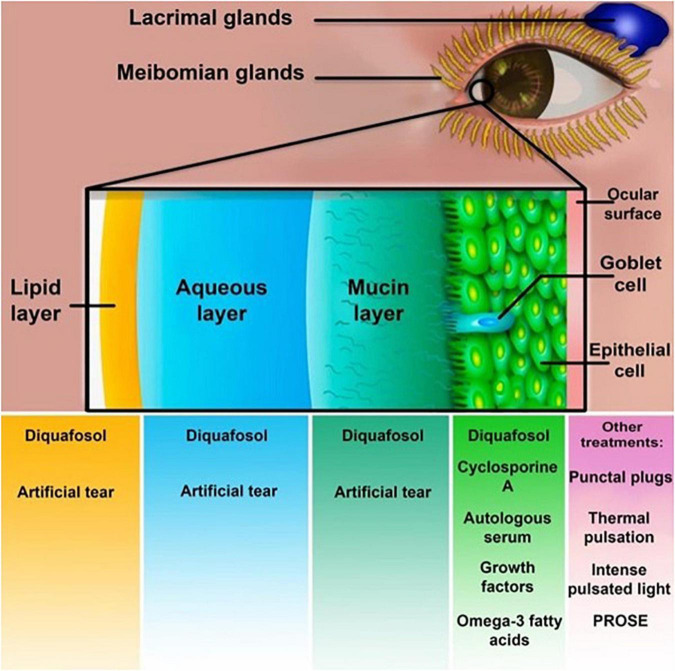
Treatment methods for post-laser-assisted *in situ* keratomileusis (LASIK) dry eye syndrome. Cyclosporine A, diquafosol, artificial tears, autologous serum, growth factors, and omega-3 fatty acids are dry eye disease (DED) treatments affecting different layers of the tear film. Punctual plugs, thermal pulsation, and prosthetic replacement of the ocular surface ecosystem (PROSE) are other treatment options.

Several studies approved that anti-nociceptive analgesics decrease post-operation chronic pain. Oral pregabalin and gabapentin are α2δ ligands used to reduce the occurrence and extent of post-surgical lingering pain following several surgeries (195). In a prospective randomized study, Galor et al. (196) demonstrated that pregabalin perioperatively administered to patients with Li-DED did not decrease the severity or frequency of their symptoms at a six-month follow-up after LASIK. Supporting previous results, Paik et al. (197) proved that pregabalin makes no reliable changes in nerve intensity or cornea sensitivity. On the other hand, we have limited information about the efficacy of pregabalin and gabapentin in treating Li-DED. Nevertheless, the existing data does not introduce them as significantly promising monotherapies (196, 197) (Supplementary Table 1). Figure 1 summarizes current treatment options for post-LASIK DED.

## Conclusion

Dry eye disease is among the most common complications in many refractive surgeries, particularly after LASIK. This disorder affects the vast majority of patients up to one year after the surgery. Moreover, DED can also turn into a chronic disease in some patients. Therefore, providing suitable methods would enhance visual results and ameliorate dryness and discomfort.

The main therapeutic options encompass punctual plugs, autologous serum, cyclosporin, artificial tear therapy, sclera lenses, and oral medication. Nonetheless, the currently existing data on the usage of pregabalin and gabapentin post-refractive surgeries needs more investigation. Overall, the most essential post-surgical approach is to provide the eye with a stable environment by increasing the production of tears and maintaining the tear surface. As a result, different types of ophthalmic drops, depicted in Figure 1, play a pivotal role in the post-surgical management of the condition. Assuming that most patients would experience Li-DED, we recommend using at least preservative-free artificial tears for a specific period, depending on the conditions of each patient. Besides, the severe or chronic forms of Li-DED may benefit from more invasive approaches such as punctual plugs. Other treatments may be beneficial, but at the surgeon’s discretion, and each treatment may be applied based on the patient’s condition. The most significant factor that predisposes patients to Li-DED is preexistent DED, which makes a preoperative assessment of each patient essential. DED management before surgery enhances wound healing and decreases the chance of DED and flap side effects. Prediction and prevention of DED, along with the provision of the right choice of treatment to each patient, is crucial to achieving better outcomes.

## Author contributions

ND: concept, design, analysis, or interpretation. AT, SE, MI, and PP: data collection or processing and literature search. AT, SE, MI, PP, AF, MP, FS, ZK, and HP: writing. All authors contributed to the article and approved the submitted version.
