# Supplementary material for: Post-LASIK dry eye disease: A comprehensive review of management and current treatment options
Source: Front Med (Lausanne). 2023 Apr 11;10:1057685. doi: 10.3389/fmed.2023.1057685 (PMC10126282; doi:10.3389/fmed.2023.1057685)
Supplement: Supplementary file 1 [file Table_1.docx]

## Supplementary Table 1: treatment approaches of post-refractive-surgery DED

| Author/ year/ ref | Type of surgery | patients/ eyes | Type of therapy | outcome | Side effects |
| --- | --- | --- | --- | --- | --- |
| Iqbal et al./ 2020  ^21^ | LASIK | 75 | Topical cyclosporine 0.05% 4t/d along with artificial tear drops for 6mo (starting 1mo after LASIK) | Mean OSDI after vs. before therapy: 21.05 ± 5.13 vs. 54.25 ± 10.81  Mean ST after vs. before therapy: 9.8 ± 1.0 vs. 5.2 ± 1.2 mm  Mean TBUT after vs. before therapy: 8.9 ± 1.1 vs. 5.6 ± 1.3 seconds  BCVA after vs. before therapy: Log MAR 0.01 ± 0.03 vs. 0.14 ± 0.09 | ___ |
| Salib et al./ 2006  ^93^ | LASIK | 21(42) | Topical cyclosporine 0.05% (T1)  artificial tears (T2)  T1 and T2 groups used artificial tears as well.  T1 and T2 were used from 1mo before LASIK to 3mo afterwards. | No remarkable difference between T1 and T2 or the base point in OSDI or BCVA  ST for T2 significantly increased 1mo after LASIK and for T1 also increased 1 week before and 1mo, 3mo and 6mo after LASIK.  No remarkable difference between T1 and T2 in uncorrected visual acuity and SPK | 3 patients in T2 group and 3 patients in T1 group experienced enhancements. |
| Kanellopoulos/ 2019  ^94^ | LASIK | 145  (145) | Topical cyclosporine A 0.05% 2 t/d for 12mo (starting 1mo after LASIK) | Mean OSDI after vs. therapy: 23.03±10.17 vs. 11.47±9.97  Mean ST after 12mo vs. before therapy: 7.6±2.0 vs. 4.5±3.6 mm  Mean TBUT after 12mo vs. before: 7.6±2.0 vs. 6.5±3 seconds | ___ |
| Eskina et al./ 2019  ^160^ | PRK | 14  (28) | Topical cyclosporine 0.05% 2 t/d for 2mo (starting 1mo after PRK) | Mean IOP after vs. before therapy: 16.2±3.21 vs. 20.5±7 mm Hg  BCVA at 6mo: 1.03±0.06  Mean UVA at 6mo: 0.98±0.05  SE at 6mo vs. before therapy: 0.04±0.12 vs. (-)5.65±1.5 Diopters | ___ |
| Torricelli et al./ 2014  ^95^ | LASIK or PRK | 642  (642) | Cyclosporine 0.05% for 1-12mo (starting 3.2 ± 2.1mo before the surgery) | After 1 year or more of therapy, 6.1% of all eyes still had DE | ___ |
| Toda et al./ 2014  ^97^ | LASIK | 105  (206) | Diquafosol tetrasodium 3% + Sodium Hyaluronate 0.3% (T1)  Diquafosol tetrasodium 3% (T2)  Artificial tears(T3)  Sodium hyaluronate 0.3% (T4)  Each 6 t/d for 4 weeks | Subjective dryness was remarkably better in T1, T2, and T4 than T3 one week after LASIK  No remarkable difference in subjective dryness of different groups 1mo after LASIK.  UVA was remarkably better in T1 than T3 and T2 after therapy  ST was remarkably better in T1 than T4 after therapy  Distance FVA only enhanced in T1  Near FVA remained the same in all groups  FSS increased remarkably in T3 with no important difference between groups  TBUT decreased remarkably in all groups with no important difference between groups  No remarkable difference in corneal sensitivity among groups  T1 is the most effective for DED symptoms after LASIK. | ___ |
| Mori et al./ 2014  ^98^ | LASIK | 15 (30) | Diquafosol 3%, 6 t/d for 12 weeks | BCVA after vs. before therapy: $-20.23$ vs. $-$20.24 log MAR  CSS in inferior cornea after 3mo of therapy vs. before therapy: (0.6 $\pm$ 0.6 and 0.7 $\pm$ 0.8) vs. (1.4 $\pm$ 0.6 and 1.3 $\pm$ 0.7)  Symptom Questionnaire results indicated significant enhancement in eye dryness and discomfort.  Mean TBUT improved remarkably but it did not change to normal.  SPK decreased significantly after therapy. | ---- |
| Wallerstein et al.  ^20^ | LASIK | 148 (296) | Artificial tear drops/  The CMC-HA free preservative group was compared with the CMC free preservative group using an average of 3-5 t/d for 3mo | Adding HA to the composition increase ocular surface epithelium recovery as well as improve visual acuity.  Visual accuracy in the CMC-HA and CMC groups before surgery was 51.1 letters and 53.1 letters, respectively, which improved by 5.8 letters and 4.7 letters on the 3mo after surgery. | ----- |
| Astakhov et al.  ^161^ | LASIK | 54 (108) | Artificial tear drops/  The preservative-free lacrimal substitute group (hylabak) was compared with the preserved lacrimal substitute group (systane) using one drop for each eye 4 t/d for 84±3 days. | The improvement in fluorescein score in the Hylabak group is probably faster than in Systane (Reduction of hyperope patients from 25.93% to 7.41% in hylabak group compared to reduction from 25.93% to 14.81% in systane group on day 28).  Patient satisfaction in the Hylabak group was significantly higher in day 28 (p=0.0113) but not in day 84 (p=0.162) | Corneal edema in 3 patients that was evaluated as a side effect of LASIK (two in the hylabak group and one in the systane group) |
| Panova et al.  ^162^ | FEMTO LASIK /  ReLEx SMILE | 20 (40) | Artificial tear drops/  The effectiveness of artificial tear drops trehalose 3% was evaluated in FEMTO LASIK and ReLEx SMILE patients for 3mo | Its use in both FEMTO LASIK and ReLEx SMILE groups reduces DED symptoms based on OSID score (10.90 ±4.5 to 8.89 ±3.26) after 3mo, increased TBUT (11.18±1.19 to 12.45±1.03 seconds) after 3mo, and improved ocular epithelium. | ---- |
| Salib et al.  ^93^ | LASIK | 21 (42) | Artificial tear drops/  Cyclosporine 0.05%/  The cyclosporine 0.05% group was compared with the unpreserved CMC 0.05% group using 2 t/d for 4mo (1mo pre-operative and 3mo postoperative). | Both treatments did not differ significantly in severity of recovery; But improvements were applied more rapidly in the cyclosporine group. | ---- |
| Zheng et al.  ^163^ | FS-LASIK | --- | Artificial tear drops /The 0.3% sodium hyaluronic group was compared with the 0.1% sodium hyaluronic group for 3mo | In both 0.3% and 0.1% sodium hyaluronic groups, NIBUT at 1 week after surgery was significantly lower than before surgery.  In the first 60,120 and 180 minutes after surgery, NIBUT of 0.1% sodium hyaluronic group did not change significantly compared to before surgery; But in the sodium hyaluronic 0.3% group, a significant change was observed in it. (p= 0.040,0.047,0.041) | ---- |
| Alio JL et al./ 2017  ^114^ | LASIK | 80 (156) | E-PRP eye drops 6 t/d as monotherapy for 6 weeks | 85% improvement in DES  93.3% improvement in Conjunctival hyperemia  71.4% improvement in at least 1 line in CDVA  CDVA after vs. before therapy: Log MAR 0.06 ± 0.12 vs. 0.14 ± 0.19 | ---- |
| Poon AC et al./  2001  ^116^ | PK | 13 (15) | Autologous serum eye drops 8 t/d for nearly 1mo | Significant improvement in 9 out of 15 eyes  Lower fluorescein score  Lower rose Bengal scores | Swelling &  redness with itch,  discharge and pain |
| Tomoko Noda-Tsuruya et al./  2006  ^164^ | LASIK | 27 (54) | Autologous serum eye drops for 1mo, 3mo, and 6mo | Lower Rose Bengal score in patients using AS at 1mo and 3mo after LASIK  TBUT increased at 3mo after LASIK  Lower fluorescein score at 1mo after LASIK | ---- |
| Shuling Pan et al.  ^165^ | LASIK | 35 (60) | Topical treatment with 1 µg/ml LIF eyedrops on the left eyes as treatment group and BSS on the right eyes as control group 4 t/d for 6mo (There was also a blank control group without any treatment) | The number of regenerated nerve fibers in the LIF group was significantly higher than that in the BSS group at all time points except the 6mo after LASIK (p<0.05). The parameters for dry eye between two groups were compared and the result showed that at 2 weeks, 1mo and 3mo after LAZIK these differences were statistically significant in the LIF group than in the BSS group (p>0.05). At 6mo, the two groups were near to the preoperative level and had no significant difference (p>0.05). | ---- |
| Myung-Jin joo et al.  ^166^ | LASIK | 16 (16) | 10 μL of topical NGF in the treatment Group and 10 μL of topical BSS in control group 4 t/d for 3 days | Eyes that were treated with topical NGF demonstrated an earlier and faster recovery of corneal sensitivity after LASIK (p=0.07). A statistically significant difference in corneal sensitivity was found between the topical NGF and control group postoperatively at 2 (p=.01), 3 (p=.03), and 4 (p=.03) weeks. | ---- |
| Sanchez-Avila et al.  ^23^ | LASIK | 42 (77) | PGRF eye drops regimen for the treatment group and a standard treatment (artificial tears, corticosteroids, AS, cyclosporine, etc.) for control group for at least 3mo along with maxifloxacin and dexamethasone 0.1%, each 4 t/d for 1 week and an artificial tear solution 4 t/d for 1mo | There were 1-4 treatment cycle with PGRF eye drops (1 cycle = 6 weeks). Results showed a statistically significant improvement in the ocular surface Disease index (38.12%), visual analogue scale scores for frequency (41.89%) and severity (42.47%), and the Schirmer test scores (88.98%) after PRGF treatment (p < 0.05). | ---- |
| Chunyan Wang et al.  ^167^ | LASIK | 42 (84) | 1 µg/ml IGF-1 eye drops for the left eyes as the treatment group and NS for right eyes as the control group by 6 t/d after LASIK | The number of corneal epithelial microvilli in the IGF-1 group was significantly higher than that in the NS group except in the second postoperative week (p < 0.05). The observation of corneal nerve regeneration showed that the number of regenerated nerve fibers in the IGF-1 group was higher at all time points (p < 0.05). The parameters of dry eye were significantly higher in the IGF-1 group compared to the control group at all time points except at 1d and 6m after LASIK. | ---- |
| Nemet et al.  ^125^ | LASIK | 345 (345) | *Intraoperative:* Insertion of punctal plugs in the lower punctum of eye at the beginning of the LASIK surgery (3mo)  *Postoperative:* dexamethasone 0.1%, non-preserved artificial tears and moxifloxacin 0.5%. Artificial tears which contained hyaluronic acid were used 5 t/d. | Patients treated using punctal plugs had obviously less postoperative dry eye complaints (N = 37 vs. 59, 21.5% vs. 34.1% respectively, P = 0.001) and patients who were treated with plugs that developed DED did so later (51.2 ± 77.64 days vs. 20.78 ± 26.9 days, P = 0.009). | ---- |
| Alfawaz et al.  ^124^ | LASIK | 39 (78) | *Intraoperative:* Insertion of punctal plugs in the lower punctum of eye (6mo), prednisolone acetate 1.0% and ofloxacin 0.3%  *Postoperative:* ofloxacin 0.3% and topical prednisolone acetate 1.0% drops 4 t/d for ten days, non-preserved artificial tear drops 4 t/d for a week | At the ultimate follow-up visit, the percentage of normal eyes was greater in eyes treated with punctal plugs for all ocular surface parameters compared to eyes were not treated with punctal plugs; nonetheless, these differences weren't statistically significant. | ---- |
| Fouda et al.  ^123^ | LASIK | 60 (120) | *Intraoperative:* Insertion of punctal plugs in the lower punctum of eye at the end of the surgery (6mo)  *Postoperative:* antibiotic eye drops in form of ofloxacin 0.3% and anti-inflammatory eye drops of fluorometholone in the first 2 weeks, preservative-free tear substitute eye drops | The punctal plug group had statistically significant increase in both TBUT (9.05 ± 1.76 s vs. 8.98 ± 2.23 s, P = 0.972) and ST score (11.04 ± 3.56 mm vs. 10.24 ± 1.91 mm, P = 0.704) with a decrease in the OSDI score (11.81 ± 3.20 vs. 12.74 ± 4.16, P = 0.552) and daily usage of lubricants (1.76 ± 2.32 vs. 2.41 ± 1.62, P = 0.588) compared to the group treated with conventional medical treatment using preservative-free tear substitutes only | Among punctal plug group, 30% of the patients complained of irritation and 30% complained of extrusion. |
| Huang et al.  ^126^ | LASIK | 8 (16) | *Postoperative:* Insertion of punctal plugs in the lower punctum of eye (1mo) | Applying punctal plugs, reductions were observed in total RMS wavefront errors (3.30 ± 1.63 μm vs. 4.16 ± 1.99 μm; P ˂ 0.01), lower-order aberration RMS (1.49 ± 1.54 μm vs. 2.80 ± 1.01 μm; P ˂ 0.01) and higher-order aberration RMS (0.50 ± 0.39 μm vs. 1.36 ± 1.30 μm; P ˂ 0.01) comparing to pre-punctal assessment. In addition, number of eyes which enjoyed visual acuity better than 20/20 improved (62.5% vs. 43.75%) comparing to pre-punctal assessment. | ---- |
| Yung et al.  ^168^ | LASIK | 18 (25) | *Postoperative:* Insertion of punctal plugs in the upper and the lower lacrimal puncta (for 2mo; from 1mo post-surgery until 3mo post-surgery) and artificial tears which contain hyaluronic acid were applied 5 t/d | Punctal plugs are able to boost not only the tear function and symptoms, but also the vision quality. The changes were significantly different between the two groups after inserting punctal plugs in the values of UCVA (plug group: -0.14 vs. -0.03, non-plug group: -0.14 vs. -0.12; P = 0.029), functional visual acuity after 10 s eye opening (FVA 10) (P = 0.021), TBUT (P = 0.008), fluorescein scores (P = 0.020), dryness and foreign body sensation (P = 0.002) in comparison to the values before insertion. Also, the rate of satisfaction with the overall outcome was significantly higher in the punctal plug group in comparison to the non-punctal plug group (P = 0.03). Schirmer values and surface regularity index after 10 s eye opening (SRI 10) didn’t change significantly. | ---- |
| Scheepers et al.  ^129^ | LASIK | 1 (2) | *Postoperative*: hourly topical lubricants (7mo), insertion of punctal plugs (7mo after the surgery), insertion of SmartPLUGs subsequently (1 year) and topical lubricants 2 t/d (1 year) | A while after starting the discomfort, the right SmartPLUGs was flushed during an irrigation and was considered responsible for the right canaliculitis. Then, she contracted left canaliculitis and the left plug was recovered using irrigations. She continued on topical lubricants and DED was cured. | 1y after the insertion of SmartPLUGs, the patient felt some ocular discomfort and after a while, she contracted bilateral canaliculitis. |
| Goyal et al.  ^169^ | LASIK | 60 (119) | 400 mg/d of vitamin E in the control group and 1.2 g/d of oral v3FA triglyceride formulation in the treatment group for 1 week pre-surgery and 12 weeks post-surgery | In patients who have LASIK surgery, v3FA Supplementation positively affects secretion of the tear. However, in comparison to the control group, v3FA Supplementation did not affect the stability of tear film. | ---- |
| Di Pascuale et al.  ^141^ | LASIK | 17 (34) | Eye-feel (Kao, Inc.), an eye-warming device, 4 t/d for 4 weeks | OSDI after vs. before therapy: 25.8 ± 18.5 vs. 60.6 ± 10.6 points  TBUT after vs. before therapy: 7.9 ± 3.6 vs. 2.4 ± 3.9 seconds  Mean lipid spread time after vs. before therapy: 0.8 ± 0.4 vs. 1.3 ± 0.4 sec  Mean lipid thickness after vs. before therapy: 79.5 ± 27 vs. 63.5 ± 23 nm  In 7 eyes, tear interference pattern changes from a vertical lipid tear deficiency to a horizontal normal | ---- |
| Schallhorn et al.  ^145^ | LASIK & PRK | 57 (109) | Thermal pulsation therapy at a mean of 40.5mo after the primary procedure | TBUT increased (difference: +1.9 sec; 1.3 to 2.5) and degree of meibomian gland dysfunction (difference: -0.69; -0.54 to -0.84) and corneal staining (difference: −0.74; −0.57 to −0.91) reduced. | ---- |
| Fuentes Páez et al.  ^170^ | LASIK refractive surgery | 11 (20) | IPL in 4 sessions (day 0, 15, 45, 75) | post-IPL vs. Pre-IPL DEWS severity grades: 1.6 ± 0.7 vs. 3.4 ± 0.5  Post-IPL vs. Pre-IPL TBUT: 5.0 ± 1.3s vs. 3.4 ± 1.6s  Post-IPL vs. Pre-IPL OSDI: 27 ± 11 vs. 34 ± 16.1 points  Post-IPL vs. Pre-IPL VA: 0.90 ± 0.15 vs. 0.67 ± 0.26 | ---- |
| Lee et al.  ^149^ | refractive surgery | 18 | Acupuncture treatment along with the usual treatment in acupuncture group for 4 weeks, a total of 12 times | OSDI score and self-assessment score of ocular discomfort decreased in acupuncture group and increased in usual care group. 4 in 7 patients showed a progress in the acupuncture treatment group. | 2 patients with moderate symptoms of upper respiratory tract infection and 1 patient with stomatitis after a 3rd molar extraction were seen in the acupuncture group. |
| Mian et al./ 2016  ^156^ | LASIK | 44 (75) | PROSE | 84% of patients indicated BCVA enhancement (P < 0.0001), PROSE was helpful in DE symptoms, corneal neuralgia, and ectasia after LASIK. | ___ |
| Galor et al./2019  ^158^ | LASIK | 42 | Pregabalin solution 150mg 2 t/d for 14 days (starting 1 day before surgery) vs control | OSDI 6mo after surgery in pregabalin group vs. before surgery: 12.6 vs. 11.9  OSDI 6mo after surgery in control group vs. before surgery: 12.3 vs. 9.0  TBUT 6mo after surgery in pregabalin group vs. before surgery: 8.35 vs. 10.9s  TBUT 6mo after surgery in control group vs. before surgery: 9.05 vs. 9.1s  ST 6mo after surgery in pregabalin group vs. before surgery: 15.45 vs. 12.8  ST 6mo after surgery in control group vs. before surgery: 15.05 vs. 17.0  Applying pregabalin peri-operatively did not indicate efficacy in reducing DED occurrence and intensity in patients observed for 6mo after LASIK. | Dizziness and fatigue were more frequent in pregabalin group than the control group 2 weeks following LASIK. |
| Paik et al./ 2020  ^159^ | LASEK | 80 (80) | Pregabalin solution 150mg 2 t/d for 15 days (starting 1 day before surgery) vs control | Pre-operative NF density in pregabalin group: 29.26; 7.66 (no/mm^2^)  NF density 6mo after LASEK in pregabalin group: 8.40; 3.10 (no/mm^2^)  Pre-operative NF density in control group: 29.14; 8.57 (no/mm^2^)  NF density 6mo after LASEK in control group: 7.70; 2.70 (no/mm^2^)  No remarkable difference between pregabalin and control groups in NF density, NF length and nerve branch density.  No remarkable difference between pregabalin and control group in cornea sensation.  During 6mo after LASEK, pregabalin was not helpful in nerve reproduction or cornea sensitivity. | 2 patients were dismissed from the study because of nausea.  Mostly moderate side effects except for the 2 dismissed patients.  No serious side effects. |

**Abbreviation**

LASIK: Laser-Assisted In Situ Keratomileusis; t/d: times per day; OSDI: ocular surface disease index; ST: Schirmer's test; TBUT: Tear film break up time; BCVA: best corrected visual acuity; SPK: superficial punctate keratitis; IOP: increasing intraocular pressure; PRK: photorefractive keratectomy; DE: dry eye;‌ UVA: uncorrected visual acuity; FVA: functional visual acuity; FSS: fluorescein staining score; CSS: conjunctival staining score; CMC: carboxymethylcellulose 0.5%; HA: hyaluronic acid; FEMTO LASIK: Femto-Laser-Assisted in Situ Keratomileusis; ReLEx SMILE: Refractive Lenticule Extraction-Small Incision Lenticule Extraction; DED: dry eye disease; FS-LASIK: Femtosecond laser-assisted laser in situ keratomileusis; NIBUT: non-invasive break-up time; E-PRP: Autologous platelet-rich plasma; DES: dry eye syndrome; CDVA: Corrected distance visual acuity; PK: penetrating keratoplasty; AS: autologous serum; LIF: leukemia inhibitory factor; BSS: balanced salt solution; NGF: nerve growth factor; PGRF: plasma rich in growth factor; IGF-1: insulin like growth factor-1; NS: normal saline; RMS: root mean square; UCVA: uncorrected visual acuity; v3FA: Omega-3 fatty acids; IPL: Intense pulsated light; DEWS: Dry Eye Workshop; VA: visual acuity; PROSE: Prosthetic Replacement of the Ocular Surface Ecosystem; NF: nerve fiber;
